# Improved outcome of HSCT in STAT1 gain-of-function disease following JAK inhibition bridging

**DOI:** 10.70962/jhi.20250027

**Published:** 2025-07-30

**Authors:** Emilie Pauline Buddingh, Mary Slatter, Juan Carlos Aldave Becerra, Laura Alonso Garcia, Erik von Asmuth, Safa Baris, Oscar de la Calle-Martín, Alice Y. Chan, Su-Wan Bianca Chan, Shanmuganathan Chandrakasan, Deepakbabu Chellapandian, Jasmeen Dara, Susan Farmand, Anders Fasth, Lisa Forbes Satter, Renata Formankova, Eyal Grunebaum, Steven J. Keogh, Ayça Kiykim, Jörn-Sven Kühl, Alexandra Laberko, Timothy Ronan Leahy, Caroline Lindemans, Caridad Martinez, Laura Martínez-Martínez, William Glenn Mitchell, Emma Morris, Joseph H. Oved, Maria Polacik, Jacques G. Rivière, Chaim M. Roifman, Sara Sebnem Kilic, Petr Sedlacek, Ami J. Shah, Linda Vong, Arjan C. Lankester, Michael H. Albert, Bénédicte Neven, Troy Torgerson, Jennifer Leiding, Catharina Schuetz, Inborn Errors Working Party (IEWP),, European Society for Blood and Marrow Transplantation (EBMT), and Primary Immunodeficiency Treatment Consortium (PIDTC)

**Affiliations:** 1 Department of Pediatrics, Pediatric Stem Cell Transplantation Program, Willem-Alexander Children’s Hospital, Leiden University Medical Center, Leiden, The Netherlands; 2 Haematopoietic Stem Cell Transplantation Department, Great North Children’s Hospital, Royal Victoria Infirmary, Newcastle, UK; 3 Allergy and Immunology, Hospital Rebagliati, Lima, Peru; 4 Pediatric Infectious Diseases and Immunodeficiencies Unit, Hospital Universitari Vall D’Hebron, Vall D’Hebron Research Institute, Barcelona, Spain; 5 Division of Pediatric Allergy and Immunology, Marmara University, Istanbul, Turkey; 6 Universitat Autònoma de Barcelona, Barcelona, Spain; 7 Department of Pediatrics, Division of Pediatric Allergy, Immunology, and Bone Marrow Transplantation, University of California, San Francisco, CA, USA; 8 Paediatric Rheumatology and Immunology Service, KK Women’s and Children’s Hospital, Singapore, Singapore; 9 Aflac Cancer & Blood Disorders Center, Children’s Healthcare of Atlanta, Emory University School of Medicine, Atlanta, GA, USA; 10 Cancer and Blood Disorders Institute, Johns Hopkins All Children’s Hospital, St. Petersburg, FL, USA; 11 Division of Pediatric Stem Cell Transplantation and Immunology, Department of Pediatric Hematology and Oncology, University Medical Center Hamburg-Eppendorf, Hamburg, Germany; 12 Queen Silvia Children’s Hospital, Gothenborg, Sweden; 13 Department of Pediatrics, Sahlgrenska Academy, University of Gothenburg, Sweden; 14 Texas Children’s Hospital, Houston, TX, USA; 15 University Hospital in Motol, Prague, Czech Republic; 16 The Hospital for Sick Children, Toronto, Canada; 17 Children’s Hospital at Westmead, Westmead, Australia; 18 İstanbul Üniversitesi-Cerrahpaşa, Istanbul, Turkey; 19 Department of Pediatrics, University Hospital Leipzig, Leipzig, Germany; 20 Department of Hematopoietic Stem Cell Transplantation, Dmitry Rogachev National Medical Research Center of Pediatric Hematology, Oncology and Immunology, Moscow, Russia; 21 Department of Paediatric Immunology, Children’s Health Ireland at Crumlin, Dublin, Ireland; 22 Department of Pediatric Immunology, University Medical Center, Utrecht, the Netherlands; 23 Hospital de la Santa Creu i Sant Pau, Barcelona, Spain; 24 University College London, London, UK; 25 Memorial Sloan Kettering Cancer Centre, New York, NY, USA; 26 Department of Paediatrics and University Center for Rare Diseases, Medizinische Fakultät Carl Gustav Carus, Technische Universität, Dresden, Germany; 27 Division of Pediatric Immunology, Bursa Uludag University, Bursa, Turkey; 28 Stanford University, Stanford, CA, USA; 29 Kinderklinik und Kinderpoliklinik, im Dr. von Haunerschen Kinderspital, Ludwig Maximilians Universität München, Munich, Germany; 30 Hopital Necker-Enfants Malades, Paris, France; 31 Allen Institute for Immunology, Seattle, WA, USA; 32 German Center for Child and Adolescent Health (DZKJ), Partner site Leipzig/Dresden, Dresden, Germany

## Abstract

Germline gain-of-function (GOF) mutations in signal transducer and activator of transcription 1 (*STAT1*) are associated with infections, including chronic mucocutaneous candidiasis and autoimmunity. Morbidity is high, and disease manifestations can be life-threatening. Curative allogeneic hematopoietic stem cell transplantation (HSCT) historically has had poor outcomes. We identified 36 patients with STAT1 GOF disease, receiving 40 HSCT procedures in 2010–2023, in a combined effort of the EBMT-IEWP and the PIDTC. Median age at first transplant was 11 years (range 1–33). Indications for HSCT were combined immunodeficiency, severe and/or refractory infections, and autoimmunity. Acute GvHD occurred in 22/40 HSCT procedures; 5 patients suffered from grade III/IV acute GvHD. One patient had chronic GvHD. Overall survival was 72.2%, and event-free survival was 55.6%, markedly improved from an earlier report on HSCT for STAT1 GOF disease. Patients with an HCT-CI score of 1 or higher had worse outcome. Pre-treatment with Janus kinase (JAK) inhibitors was associated with better event-free survival.

## Teaser

STAT1-GOF disease is characterized by infections and autoimmunity. In this article, overall survival after allogeneic HSCT in an international cohort of 36 patients is 72.2%, markedly improved from an earlier report. Pre-treatment with JAK inhibitors is associated with better event-free survival.

## Introduction

Autosomal dominant gain-of-function (GOF) mutations in signal transducer and activator of transcription 1 (*STAT1*) were identified in 2011 as a cause of chronic mucocutaneous candidiasis (1, 2). Since then, it has been recognized that STAT1 GOF disease is an inborn error of immunity with severe immune dysregulation and various effects on humoral and adaptive immunity that lead to increased susceptibility to a variety of bacterial, viral, and fungal pathogens. The occurrence of vascular aneurysms and carcinoma are associated with particularly poor outcome (3). Hyperinflammation and organ-specific and systemic autoimmunity are also a key feature of STAT1 GOF disease. This is mediated through increased signaling through the Janus kinase (JAK)/STAT pathway and an enhanced response to cytokine signaling, including type I interferons (IFNs) (4). Emerging reports show promising results with the use of JAK inhibitors to treat STAT1 GOF disease (5, 6, 7, 8, 9, 10, 11, 12, 13, 14, 15, 16). However, it requires careful balancing to prevent severe infectious complications (17). In addition, not all patients have complete remission of symptoms, and it is not yet clear if prolonged treatment with these agents will result in sustained long-term disease control and better outcome (7).

Early reports of hematopoietic stem cell transplantation (HSCT) in STAT1 GOF disease, which was often performed in patients with severe disease manifestations, had mixed outcomes (6, 16, 18, 19, 20, 21, 22). In the largest series of transplanted patients with STAT1 GOF disease reported thus far (*N* = 15) (18), there was an abysmal overall survival (OS) of 40% and an event-free survival (EFS) of only 10%, mainly related to high rates of secondary graft failure. Although not completely understood, the hyperinflammatory state associated with the enhanced response to circulating cytokines, such as IFNs, is thought to contribute to the poor outcome of HSCT for STAT1 GOF disease, as is also seen in other IFN-driven diseases (23). Because JAK inhibition for treatment of STAT1 GOF disease is effective in controlling immune dysregulation, treatment with JAK inhibitors has been increasingly used as a “bridge” to HSCT to optimize clinical condition prior to HSCT. In a recent report on JAK inhibitor treatment in patients with STAT1 or STAT3 GOF disease, transplanted patients pre-treated with JAK inhibitors had an excellent OS of 91% (7).

Here, we report the latest outcome data of patients transplanted for STAT1 GOF disease from an international cohort representing 14 countries and give an update on the historical cohort in this joint Primary Immune Deficiency Treatment Consortium (PIDTC)/European Society for Blood and Marrow Transplantation – Inborn Errors Working Party (EBMT-IEWP) retrospective study.

## Results

### Patients

**Figure S1.**
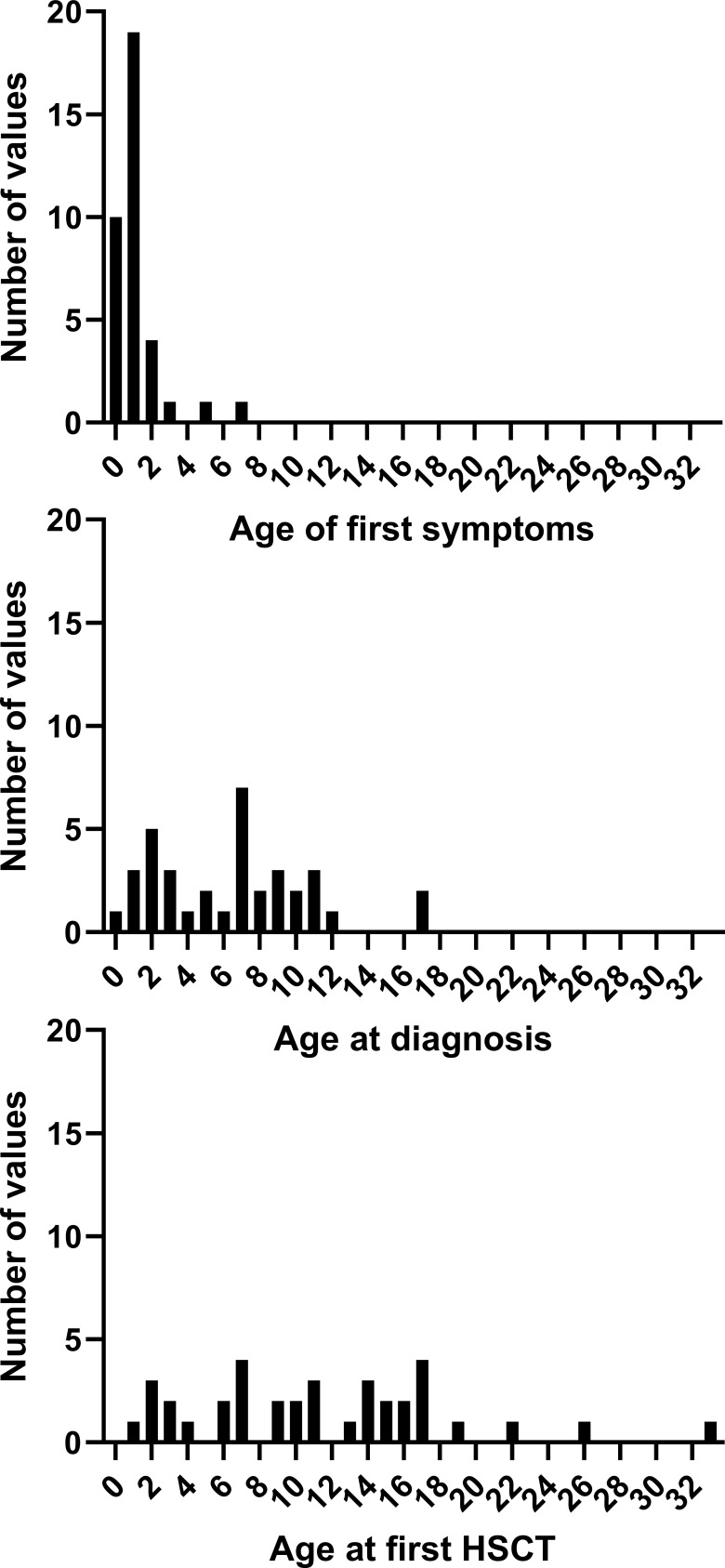
Age of first symptoms, age at diagnosis, and age at first HSCT.

36 patients with STAT1 GOF disease who underwent HSCT since 2010 were included (Table 1). Range of follow-up of surviving patients is 114 days–8 years. 15 patients (42%) were female and 21 (58%) were male. Data of 7 patients previously reported by Leiding et al. (18) were included (with follow-up clinical data if still alive) in the new eCRF to allow for an integral analysis. Patients reported in other manuscripts are also shown in Table 1. 20 patients had defects in the DNA-binding domain, 14 in the coiled-coil domain (CCD), and one each in the SH2 or TAD domain. The median age at first symptoms of STAT1 GOF disease was 0.8 years (interquartile range [IQR] 0.4–1 year), the median age at diagnosis was 7 years (IQR 2.25–9.3 years), and the median age at first HSCT was 11 years (IQR 6.25–16 years) (Fig. S1).

**Table 1.** Patients and transplant characteristics

Patient	HSCT year	STAT1 variant (protein)	STAT1 mutation protein domain	Donor (match)	Graft source	Conditioning group	Conditioning agents	Previously reported (reference)
1*	2019	E353K	DBD	MSD	BM	MAC	ATG; BU iv 14 mg/kg; Flu 160 mg/m^2^	​
2	2019	V653I	SH2D	MUD (10/10)	BM	MAC	ATG; Treo 42 g/m^2^; Flu 150 mg/m^2^; TT 8 mg/kg	(24)
3* HSCT 1	2021	K388E	DBD	MUD (10/10)	BM	MAC	Alemtuzumab; Treo 42 g/m^2^; Flu 150 mg/m^2^; TT 8 mg/kg	(7)
3 HSCT 2	2023	K388E	DBD	MUD (10/10; 12/12)	BM	MAC	Alemtuzumab; BU iv (target AUC 90 mg*h/l); Flu 160 mg/m^2^	​
4*	2023	R274W	CCD	MUD (10/10; 12/12)	BM	MAC	Alemtuzumab; Treo 42 g/m^2^; Flu 150 mg/m^2^; TT 8 mg/kg	​
5	2021	P293L	CCD	MUD (10/10)	BM	MAC	ATG; Treo 42 g/m^2^; Flu 160 mg/m^2^; TT 10 mg/kg	​
6	2020	T385M	DBD	MUD (9/10)	BM	MAC	ATG; Treo 42 g/m^2^; Flu 160 mg/m^2^; TT 10 mg/kg	​
7*	2015	S466R	DBD	MUD (10/10; 11/12)	PBSC	Myeloablative, reduced intensity	Alemtuzumab; Treo 42 g/m^2^; Flu 150 mg/m^2^	(18)
8*	2018	L283S	CCD	MUD (10/10; 11/12)	PBSC	Myeloablative, reduced intensity	Alemtuzumab; Treo 42 g/m^2^; Flu 150 mg/m^2^	(25)
9*	2018	E705Q	TAD	MUD (10/10; 12/12)	PBSC	Myeloablative, reduced intensity	Alemtuzumab; Treo 42 g/m^2^; Flu 150 mg/m^2^	(15)
10 HSCT 1	2014	T385M	DBD	MUD (10/10)	PBSC; αß-T cell depletion	Myeloablative, reduced intensity	ATG; Treo 42 g/m^2^; Flu 150 mg/m^2^; rituximab 375 mg/m^2^	(18)
10 HSCT 2	2014	​	​	ORD (5/10)	PBSC; αß-T cell depletion	MAC	ATG; CY 120 mg/kg; Flu 150 mg/m^2^; melphalan 140 mg/m^2^; rituximab 100 mg/m^2^	​
11*	2018	K388R	DBD	ORD (6/10)	PBSC(T-αß depletion)	MAC	ATG; Treo 42 g/m^2^; Flu 150 mg/m^2^; TT 10 mg/kg; rituximab 100 mg/m^2^	​
12	2022	T385M	DBD	MSD	PBSC	MAC	Alemtuzumab; Treo 42 g/m^2^; Flu 180 mg/m^2^; TT 10 mg/kg	​
13*	2020	R274Q	CCD	MUD (10/10)	PBSC	MAC	Alemtuzumab; Treo 42 g/m^2^; Flu 150 mg/m^2^; TT 10 mg/kg	​
14*	2021	R274Q	CCD	MUD (10/10)	BM	MAC	Alemtuzumab; Treo 42 g/m^2^; Flu 150 mg/m^2^; TT 10 mg/kg	​
15	2021	I294T	CCD	MUD (9/10)	BM	Myeloablative, reduced intensity	ATG; Treo 42 g/m^2^; Flu 150 mg/m^2^	​
16* HSCT 1	2021	T288P	CCD	MUD (9/10)	BM plus CD34^+^-selected PBSC	MAC	ATG; BU iv (target AUC 70 mg*h/l); Flu 160 mg/m^2^; TT 5 mg/kg	(6, 7)
16 HSCT 2	2021	​	​	ORD (5/10)	PBSC	Non-myeloablative	Alemtuzumab; CY 29 mg/kg; Flu 150 mg/m^2^; rituximab 375 mg/m^2^	​
17*	2017	K344E	DBD	MSD	BM	MAC	Alemtuzumab; BU iv 12.8 mg/kg; Flu 180 mg/m^2^	(6, 7)
18*	2020	L206P	CCD	MUD (9/10)	PBSC	MAC	ATG; Treo 42 g/m^2^; Flu 150 mg/m^2^; TT 10 mg/kg	​
19*	2019	T385M	DBD	MUD (10/10)	BM	MAC	ATG; Treo 42 g/m^2^; Flu 150 mg/m^2^; TT 10 mg/kg	(16)
20	2016	T385M	DBD	MSD	CB	Myeloablative, reduced intensity	ATG; Treo 42 g/m^2^; Flu 160 mg/m^2^	(19)
21	2015	C324F	DBD	MUD (10/10)	BM	Myeloablative, reduced intensity	ATG; Treo 42 g/m^2^; Flu 150 mg/m^2^	(19)
22	2022	R210K	CCD	ORD (5/10)	BM	MAC	ATG; BU iv (target AUC 90 mg*h/l); Flu 160 mg/m^2^; emapalumab 12 mg/kg	(20)
23*	2021	T419R	DBD	MSD	BM	MAC	ATG; melphalan 140 mg/m^2^; Flu 150 mg/m^2^; TT 10 mg/kg	(7)
24*	2021	T385M	DBD	MUD (10/10)	BM	Non-myeloablative	ATG; melphalan 140 mg/m^2^; Flu 160 mg/m^2^; TT 10 mg/kg; rituximab 375 mg/m^2^	​
25*	2018	T385M	DBD	MUD (9/10)	PBSC	Non-myeloablative	ATG; melphalan 140 mg/m^2^; Flu 160 mg/m^2^; TT 10 mg/kg; rituximab 375 mg/m^2^	​
26	2022	R210K	CCD	MSD	BM	Myeloablative, reduced intensity	ATG; BU iv 71.75 mg*h/l; Flu 160 mg/m^2^; rituximab 375 mg/m^2^	(7)
27	2013	T385M	DBD	MSD	BM (CD3^+^ depleted)	MAC	BU iv 16 mg/kg; CY 200 mg/kg	(18, 21)
28	2013	N397D	DBD	MUD (10/10)	BM	MAC	BU iv 14.4 mg/kg; CY 120 mg/kg; etoposide 30 mg/m^2^	(18)
29*	2020	T385K	DBD	MSD	PBSC(T-αß depleted)	Non-myeloablative	ATG; melphalan 100 mg/m^2^; clofarabine 40 mg/m^2^; TBI 200 cGy; rituximab 200 mg/m^2^	​
30	2011	N397D	DBD	MSD	BM	Non-myeloablative	ATG; melphalan 140 mg/m^2^; Flu 150 mg/m^2^	(18, 22)
31	2021	R274W	CCD	MUD (9/10)	PBSC	MAC	ATG; melphalan 140 mg/m^2^; Flu 150 mg/m^2^; Cy 150 mg/kg	​
32	2010	R274W	CCD	MSD	BM	Non-myeloablative	None	(18)
33 HSCT 1	2014	D165G	CCD	UCB (9/10)	CB	MAC	ATG; BU iv (target AUC 90 mg*h/l); Flu 160 mg/m^2^	(18)
33 HSCT 2	2014	D165G	CCD	MUD (10/10)	CB	Myeloablative, reduced intensity	Treo 42 g/m^2^; Flu 160 mg/m^2^	​
34*	2019	V389G	DBD	MUD (9/10)	BM	MAC	Alemtuzumab; BU iv 9.6 mg/kg; Flu 175 mg/m^2^; TT 10 mg/kg; CY 100 mg/kg	​
35	2020	C324Y	DBD	MUD (9/10)	PBSC	MAC	ATG; Treo 42 g/m^2^; Flu 150 mg/m^2^; TT 10 mg/kg	(7)
36*	2020	M202V	CCD	MUD (10/10)	PBSC	MAC	Alemtuzumab; melphalan 140 mg/m^2^; Flu 150 mg/m^2^; TT 8 mg/kg	(7)

ATG; antithymocyte globulin; BU, busulfan; CB, cord blood; CY, cyclophosphamide; DBD, DNA-binding domain; Flu, fludarabine; MAC, myeloablative conditioning; MSD, matched sibling donor; MUD, matched unrelated donor; ORD, other related donor; PBSC, peripheral blood stem cells; SH2D, Src homology 2 domain; TAD, transactivation domain; TBI, total body irradiation; Treo, treosulfan; TT, thiotepa. Patients with * received ruxolitinib prior to their (first) transplant.

### Disease burden prior to HSCT

**Figure S2.**
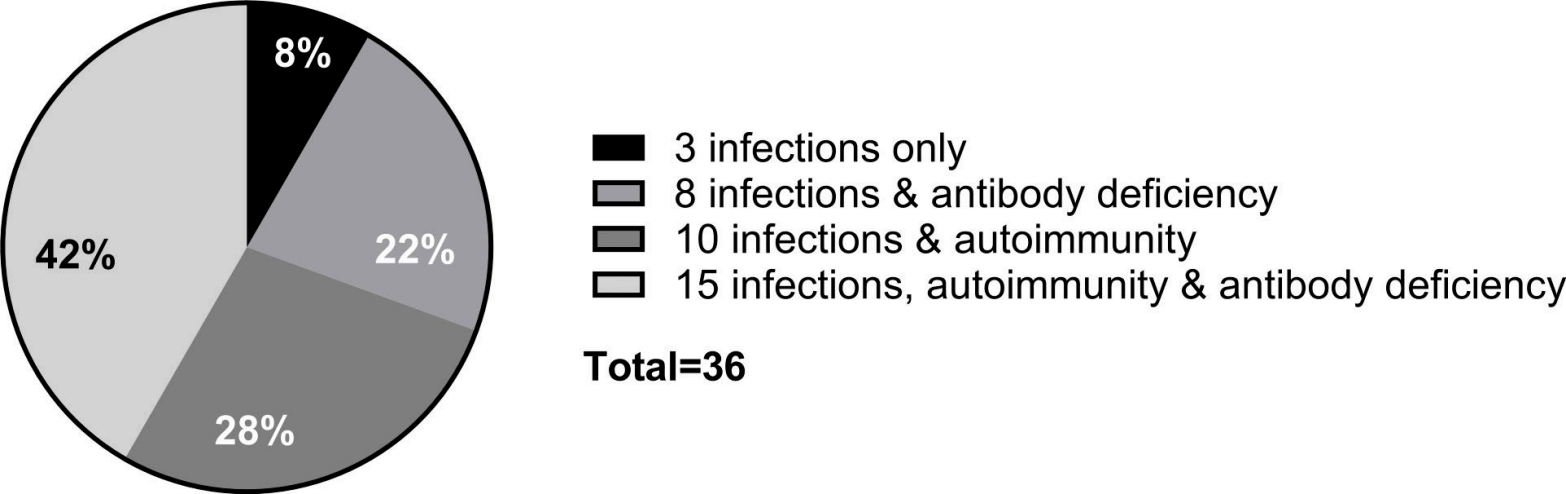
Presence of infections, autoimmunity, and antibody deficiencies in STAT1 GOF patients prior to first HSCT.

All patients had infections prior to first HSCT, with a median age of first infection of 0.8 years (Table S2 and Fig. S2). Mucocutaneous fungal infections were most common, in 34/36 (94%) patients. Nine of 36 patients (25%) had invasive fungal infections. Other common infections were skin and lung infections, often caused by *Staphylococcusaureus*, in 11/36 patients (30.5%). Autoimmune disease was present in 25/36 (69%) patients, with a median age of onset of 3.7 years (Table S3), most commonly thyroid disease and autoimmune cytopenia. Immunosuppressive medication was prescribed at any time prior to first HSCT in 26/36 (72%) patients, most commonly steroids and/or a JAK inhibitor. Two patients had hemophagocytic lymphohistiocytosis, both at 10 years of age. Two-thirds of patients had an antibody deficiency (Table S4), and immunoglobulin replacement therapy was prescribed prior to first HSCT in 25/36 (69%) patients. Two-thirds of patients (22/36, 61%) had a history of failure to thrive. Many patients had chronic lung disease, including bronchiectasis (15/36, 42%). Four patients (11%) had aneurysms prior to first HSCT, at a median age of 12.5 years (range 0.9–35 years): three cerebral and one extracerebral aneurysm. There were no patients with malignancies in this cohort. Most patients (69%) had an hematopoietic cell transplantation-specific comorbidity index (HCT-CI) score of 1 or higher prior to first HSCT, all but two patients had been hospitalized at least once, and only 8/36 (22%) had a Karnofsky (adults) or Lansky (children) performance status of 100 (Table S5).

### Ruxolitinib treatment prior to HSCT

20 (56%) patients were treated with ruxolitinib leading up to their first HSCT. One patient received baricitinib in the past, but not directly prior to HSCT. Ruxolitinib was started a median of 6 mo prior to the first HSCT (IQR 4–18 mo). A wide range of doses was administered, with a median of 15 mg/m^2^/day (IQR 9–26 mg/m^2^/day). 13 of the 20 ruxolitinib-pre-treated patients (65%) received concomitant fluconazole, itraconazole, voriconazole, or posaconazole, which are presumed to increase exposure to ruxolitinib by about twofold. Ruxolitinib was stopped in 18 patients directly prior to start of the conditioning regimen. One patient continued ruxolitinib for graft versus host disease (GVHD) prophylaxis after the infusion of the stem cells, and time of discontinuation is unknown for one patient.

### HSCT characteristics

There were 40 transplant procedures in 36 patients (Table 1). Indications for first HSCT were combined immunodeficiency in 14/36 (39%), autoimmunity and/or IPEX-like disease in 10/36 (28%), and severe infections in 12/36 (33%) of patients. Combined immunodeficiency was characterized as quantitative or functional defects in humoral and adaptive immunity; IPEX-like disease was a predominant presence of autoimmunity and hyperinflammation. Indications for re-transplant were graft failure (*n* = 3, one with primary graft failure and two with secondary graft failure) or relapse (*n* = 1, decreasing donor chimerism with recurrence of STAT1 GOF symptoms). Most patients received myeloablative conditioning (24/40 or 60% of transplant procedures) or myeloablative reduced-intensity conditioning (10/40 transplant procedures or 25%). In 6/40 transplant procedures, non-myeloablative conditioning was given because of poor clinical condition of the patient, recent transplant with graft failure, and/or very young age. Serotherapy was administered as part of the conditioning regimen in 36/40 (90%) HSCT procedures (ATG *n* = 23, alemtuzumab *n* = 13). Graft source was unmanipulated bone marrow (BM) in 20/40 (50%) HSCT procedures, PBSC in 15/40 (38%), of which 4 were CD3+TCR alphabeta depleted, CB in 3/40 (8%), and manipulated BM in 2/40 (5%). A calcineurin inhibitor plus mycophenolate mofetil (13/40) or methotrexate (12/40), with or without other agents, were the most commonly used GVHD prophylaxis regimens (Table S6). In four HSCT procedures, posttransplant cyclophosphamide was used, all in a mismatched donor setting.

### HSCT outcome: Engraftment, chimerism, and GVHD

**Figure S3.**
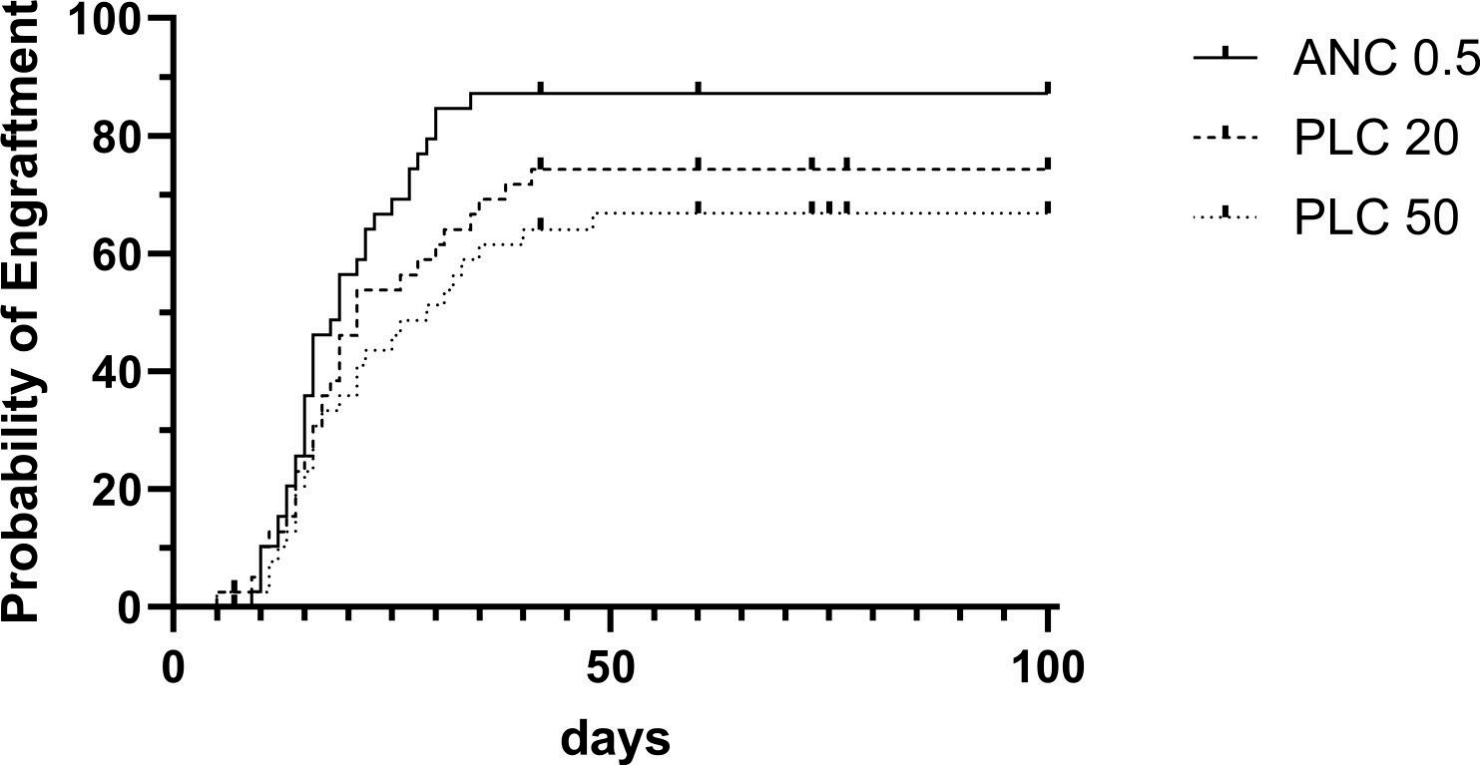
**Engraftment of neutrophils and platelets.** Kaplan–Meier curve of engraftment. Events defined as: ANC 0.5, neutrophil engraftment of >/=0.5*10e6/L, first of 3 consecutive values; PLC 20, platelet engraftment (>/=20*10e9/L, first of 3 consecutive values without transfusion); PLC 50, platelet engraftment (>/=50*10e9/L, first of 3 consecutive values without transfusion). Data censored at death, re-transplant, or no engraftment at day +100 (whichever comes first).

Probability of achieving engraftment by 100 days after HSCT were 87.2% for absolute neutrophil count (ANC) 0.5, 74.3% for platelet count (PLC) 20, and 66.8% for PLC 50 (Fig. S3). After the first HSCT, 24/36 (64%) of patients achieved durable full donor chimerism (Table S7). Overall, 27/40 (68%) transplant procedures resulted in full donor chimerism at last follow-up (Table S8). Acute GVHD occurred after 18/40 (45%) HSCT procedures, but only 6/40 (15%) were grade III or IV. Chronic GVHD (liver) occurred in one patient.

### HSCT outcome: Survival

**Figure S4.**
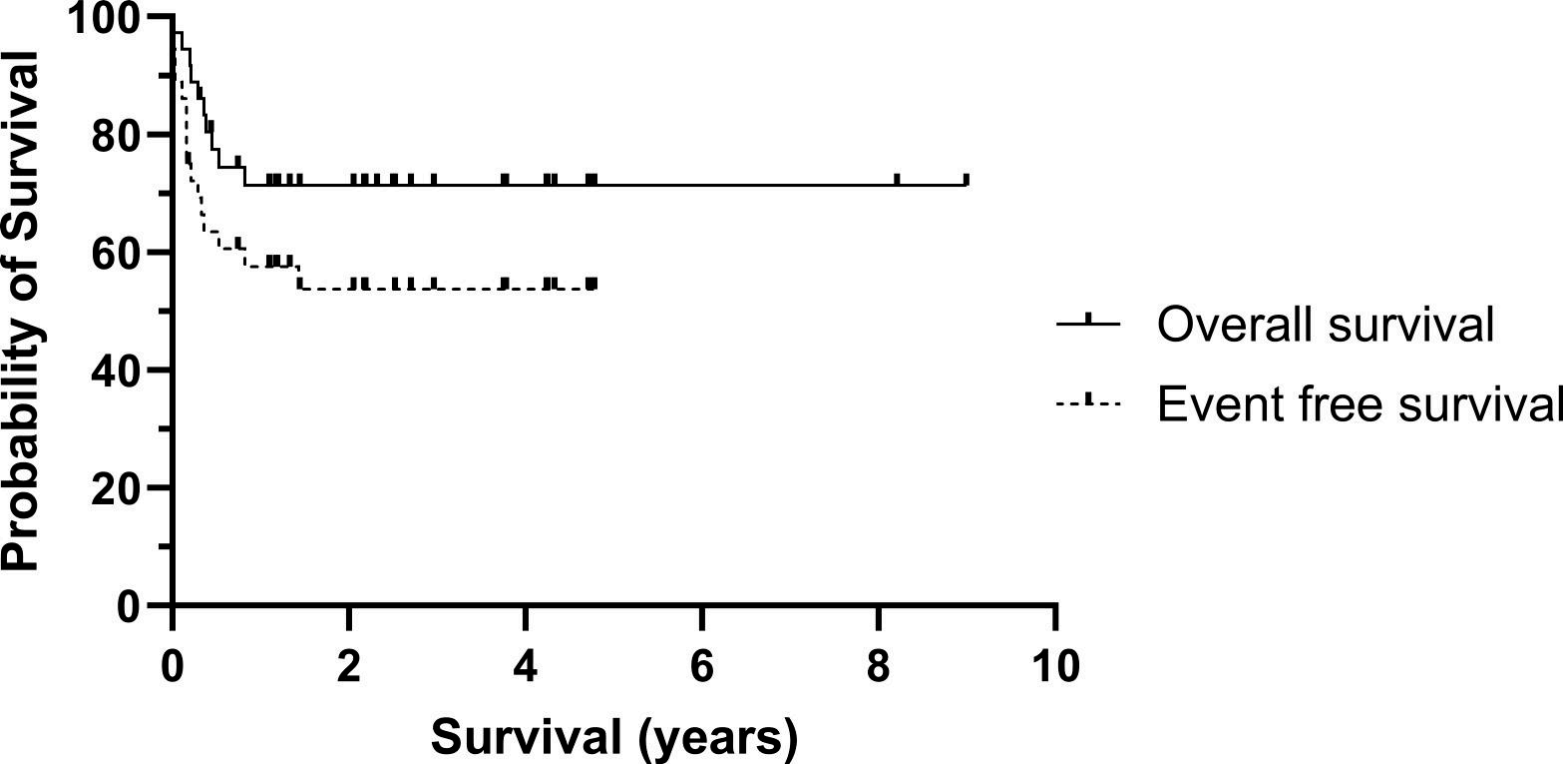
**Kaplan–Meier survival curve of OS and EFS.** For OS, events are defined as death from any cause, and patients are censored at last date of follow-up. For EFS, events are defined as graft failure, re-transplant, acute GVHD grade III or IV, moderate to severe chronic GVHD, or death of any cause, whichever occurred first.

OS of this cohort was 72.2%, and EFS was 55.6% (Fig. S4). Causes of death were sepsis with HLH in one patient, pulmonary infection in one patient, likely pulmonary transplant-associated thrombotic microangiopathy in one patient, intracranial hemorrhage due to cerebral aneurysm in one patient, infection with inflammatory pneumonitis in one patient, immune cytopenia with infection in one patient, graft failure with infection in two patients, and acute GVHD with multi-organ failure in two patients. In Table 2, the outcomes of all individual patients are listed. There was no significant difference in OS according to age at transplantation (above or below the median of 11 years).

**Table 2.** Patient outcome

Patient number	Outcome at last follow-up	Days of follow-up (since 1st HSCT)
1	Full donor, alive and well	987
2	Died (aGVHD), full donor	Died at day +139
3	Re-HSCT, alive and well, full donor	849
4	Full donor, alive and well	114
5	Full donor, alive and well	485
6	aGVHD gr IV (alive and well), full donor	913
7	Full donor, alive and well	1,747
8	Full donor, alive and well	525
9	Alive and well, mixed donor	1,551
10	Re-HSCT, alive and well, full donor	3,000
11	Full donor, alive and well	1,370
12	Graft failure, cGVHD, full donor	160
13	Full donor, alive with bronchiectasis	799
14	Full donor, alive and well	428
15	Full donor, alive and well	791
16	Graft failure, after second HSCT full donor, poor immune reconstitution	440
17	Alive with bronchiectasis, mixed donor	1,719
18	Died (cytopenia and infection), full donor	Died at day +75
19	Full donor, alive and well	1,380
20	Full donor, alive and well	1,550
21	Died (graft failure and infection), complete autologous reconstitution	Died at day +106
22	Full donor, alive and well	435
23	Full donor, alive and well	400
24	Died (pulmonary transplant-associated microangiopathy), full donor	Died at day +130
25	Died (aGVHD and multi-organ failure), full donor	Died at day +73
26	Full donor, alive and well	270
27	aGVHD gr IV (currently alive with kidney failure), full donor	3,285
28	Died (refractory HLH with sepsis and multi-organ failure), full donor	Died at day +42
29	Full donor, alive and well	920
30	Died (respiratory insufficiency), mixed chimerism	Died at day +299
31	Died (graft failure and infection), full donor	Died at day +193
32	Died (intracranial bleeding), chimerism non-evaluable	Died at day +7
33	Died (graft failure, re-HSCT, infection, and pneumonitis), full donor	Died at day +166
34	Full donor, alive and well	1,582
35	Full donor, alive and well	1,080
36	Full donor, alive and well	749

**Figure 1.**
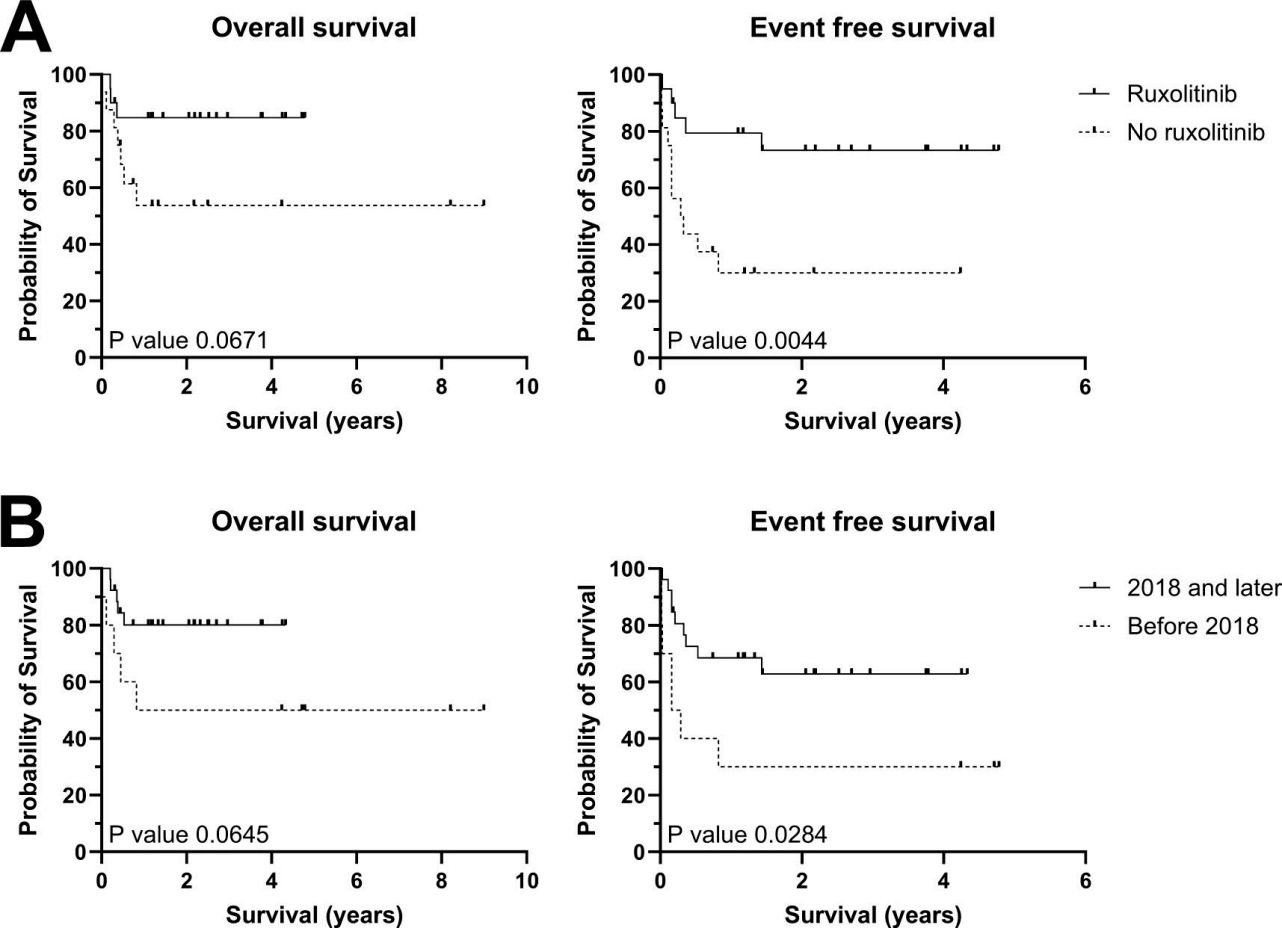
**OS and EFS stratified by ruxolitinib pre-treatment and calendar year of HSCT. (A)** The patients that were treated with ruxolitinib leading up to the first HSCT had significantly better EFS and a trend for better OS. **(B)** Patients receiving their first transplant in 2018 or later had better EFS and a trend for better OS. Kaplan–Meier survival curves with log-rank P values. For OS, events are defined as death from any cause, and patients are censored at last date of follow-up. For EFS, events are defined as graft failure, re-transplant, acute GVHD grade III or IV, moderate to severe chronic GVHD, or death of any cause, whichever occurred first.

In univariate analyses, pre-treatment with ruxolitinib was associated with a trend for better OS (P 0.0671) and was significantly associated with better EFS (P 0.0044) (Fig. 1). OS was 84% in the ruxolitinib-treated group compared with 54% in the untreated group, and event survival was 73% versus 30%, respectively. Similarly, there was a trend for patients transplanted after 2018 to have a better OS (P 0.0645) and a significantly better EFS (P 0.0284). In a multivariate Cox regression analysis with both ruxolitinib and alemtuzumab and HSCT before or after 2018 as covariates, only ruxolitinib pre-treatment was significantly associated with better EFS (HR 0.286, 95% CI 0.092–0.894, P = 0.031). There was no significant difference in time to engraftment of neutrophils or platelets and no difference in the number achieving full donor first chimerism between those pre-treated with ruxolitinib and those not treated.

**Figure S5.**
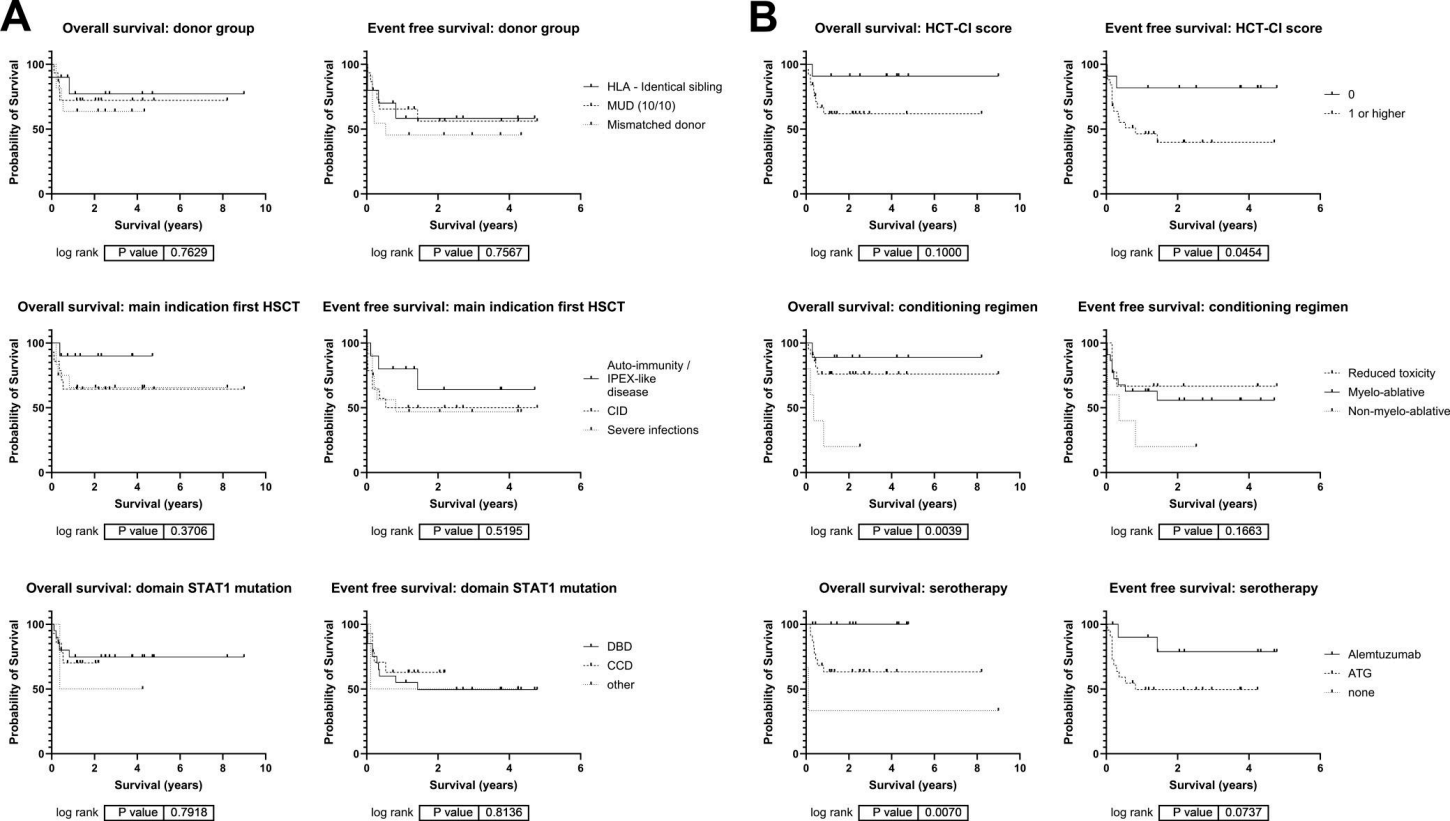
**Kaplan–Meier OS and EFS curves, stratified by baseline characteristics and treatment. (A and B)** Kaplan–Meier OS and EFS curves, stratified by (A) donor group, indication for first HSCT, and domain of STAT1 mutation, and (B) HCT-CI score, conditioning regimen, and serotherapy, with log-rank P values. For OS, events are defined as death from any cause, and patients are censored at last date of follow-up. For EFS, events are defined as graft failure, re-transplant, acute GVHD grade III or IV, moderate to severe chronic GVHD, or death of any cause, whichever occurred first.

There were no differences in OS or EFS for patients with HLA-identical versus unrelated matched or mismatched donors, primary indication for HSCT (autoimmunity/IPEX-like disease, CID, or severe infections), or STAT1 mutation domain (BDB domain, CCD, or other) (Fig. S5 A). Patients with a HCT-CI score of 1 or higher had worse EFS (P 0.0454) and a trend for worse OS (P 0.10) (Fig. S5 B). Patients receiving myeloablative conditioning regimens had similar outcome as patients receiving reduced-intensity myeloablative conditioning regimens. Patients receiving non-myeloablative conditioning had worse event-free and OS. 2 of the 18 patients in the ATG group received non-myeloablative conditioning versus 0/12 in the alemtuzumab group, but this difference was not statistically significant (Fisher’s exact test P value 0.50). Patients receiving alemtuzumab had better OS and EFS as compared with patients receiving ATG or no serotherapy (P 0.0070 and 0.0737, respectively).

## Discussion

Allogeneic HSCT is a curative treatment for STAT1 GOF disease, but experience is still limited, in part because historical data on outcome of HSCT for this condition were discouraging. In the previous cohort study from Leiding et al. (18), many patients suffered from secondary graft failure, and OS was only 40%. Here, we analyzed data from patients with STAT1 GOF disease transplanted from 2010–2023. Data from patients previously reported were also reentered, to allow for an integral analysis. Most patients in the current study were severely affected. All patients had infections, the majority had autoimmunity, many had chronic lung disease and failure to thrive, and all but two patients had been hospitalized at least once prior to HSCT. Despite this high burden of disease, outcome after HSCT was markedly improved as compared with the previous report, with 72.2% OS and 55.6% EFS.

Targeted therapies such as JAK inhibitors are now accessible in many countries worldwide and allow for better control of inflammatory symptoms with few medication side effects when modulating the JAK-STAT pathway in patients with STAT1 GOF disease (5, 7). Despite these therapeutic advances, long-term experience with JAK inhibition in this rare inborn error of immunity is still missing. Transplanted patients that were pre-treated with the JAK inhibitor ruxolitinib had significantly better EFS than patients not receiving ruxolitinib. Also, there was a trend for better OS.

In this cohort, posttransplant infections, with or without cytopenia or graft failure, were an important cause of death, in 6/10 deceased patients. Two patients died because of complications of severe acute GVHD. Six of 40 HSCT procedures (15%) resulted in graft failure. Although patients receiving a transplant after 2018 and those receiving alemtuzumab also had better outcome, multivariate analysis, including these possible confounders as covariates, showed that only ruxolitinib pre-treatment was significantly associated with better EFS. We hypothesize that the better EFS in patients pre-treated with JAK inhibitors is due to a better control of immune dysregulation prior to transplant, resulting in less graft failure and less inflammatory complications posttransplant in patients treated with a JAK inhibitor. Perhaps this is at least partly mediated through a reduction in IFN-γ levels peri-transplant, thereby preventing IFN-γ-mediated graft failure (23). Another way to reduce IFN-γ levels is by adding the anti-IFNγ antibody emapalumab to the conditioning regimen, which was done in one patient in the current cohort (patient 22). This patient is currently doing well, over a year post-HSCT (20).

At last follow-up, 21/36 patients (58%) were alive and well, and 5/36 (14%) were alive with complications: poor immune reconstitution in one, kidney failure in one, bronchiectasis in two (previously present in 1, but not the other patient), and chronic GVHD with graft failure in one. Most surviving patients had full donor chimerism. One of the surviving patients with mixed chimerism was alive and well at last follow-up; the other surviving patient with mixed chimerism was alive with bronchiectasis. Because most patients in this study had full donor chimerism at last follow-up, we were not able to assess if mixed donor chimerism is sufficient to control STAT1 GOF disease symptoms.

Patients receiving non-myeloablative conditioning regimens had worse outcome than patients receiving (reduced intensity) myeloablative regimens. This is probably confounded by the fact that patients with the highest morbidity were not in a clinical condition to receive more intensive conditioning. Patients receiving alemtuzumab had better OS and EFS than patients receiving ATG or no serotherapy, perhaps because of better control of peri-transplant inflammation. Donor match grade was not associated with OS or EFS, although the numbers of patients in each donor group were small. Similarly, there were no differences in outcome depending on the primary indication for HSCT or which protein domain was affected by the STAT1 mutation.

Due to improved and accessible genetic diagnostics in clinical practice, patients with inborn errors of immunity, such as STAT1 GOF disease, can be diagnosed earlier following symptom onset. Here, we show that those STAT1 GOF patients who had most disease-related complications and therefore higher scores on the HCT-CI had worse EFS and a trend for worse OS. However, it remains difficult to determine the optimal timing for HSCT in these patients. As in other inborn errors of immunity, the choice for or against curative cell therapy depends on the clinical condition of each patient with the aim not to miss a window of opportunity in which this therapeutic option can be safely offered. Here lies the dilemma for patients and treating physicians: the outcome of HSCT is usually better when the disease is not yet advanced, while transplant-associated complications increase and are more difficult to predict in patients with permanent or progressive organ dysfunction, such as chronic lung or liver disease or chronic infections. Also, our data show that pretransplant control of immune dysregulation improves the\ outcome of patients with STAT1 GOF disease undergoing HSCT, which may also be true for other inborn errors of immunity with immune dysregulation.

In conclusion, allogeneic HSCT is a curative and safe treatment option for patients with STAT1 GOF disease, particularly for those patients without significant comorbidities. Pre-HSCT use of JAK inhibitors is recommended to control disease-related symptoms, reduce the possibility of graft failure, and improve survival.

## Materials and methods

### Patients

We included 36 patients with STAT1 GOF disease who underwent HSCT regardless of outcome after 2010 from PIDTC and EBMT-IEWP centers. Inclusion was also solicited by announcement through the EBMT-IEWP and the PIDTC as well as by contact to centers of all published cases. Informed consent of participants was obtained according to the local regulations of the participating centers, in accordance with the Declaration of Helsinki.

### Data collection

Data were collected via Castor Electronic Data Capture (26), using a shared eCRF. Basic demographic data included age, sex, genetic diagnosis, age at diagnosis, age at HSCT, time from diagnosis to HSCT, comorbidities, and year of HSCT. Transplant-specific demographics included donor type, cell source, conditioning intensity, and use of serotherapy. We also collected data on prior usage of immunosuppressive treatment, including treatment with JAK inhibitors. We collected data on HSCT complications, outcome including clinical condition at last follow-up, and donor chimerism. An attempt was made to obtain follow-up clinical data from the patients reported by Leiding et al. (18) and for those transplanted after 2010, a request was made to reenter data in the current eCRF (Table S1).

### Definitions

For all patients, pretransplant disease burden was assessed by calculating the HCT-CI (27). The conditioning regimen was classified as myeloablative (regimen A or B from the EBMT/ESID IEWP guidelines paper [28]), myeloablative reduced intensity (regimen C or D), or reduced intensity/non-myeloablative (regimen E or F). Neutrophil engraftment was defined as the first of three consecutive days with an ANC of 0.5*10^6^/L or higher (ANC 0.5). Thrombocyte engraftment was defined as the first of three consecutive days with PLCs of 20*10^9^/L (PLC 20) or 50*10^9^/L (PLC 50), without transfusion support for the last 7 days. Chimerism was reported as either whole-blood or lineage-specific analysis as per center protocol. Full donor chimerism was defined as >95% donor chimerism, mixed chimerism as 5–95% donor chimerism, and complete autologous reconstitution as >95% patient chimerism. For grading of acute GVHD, the modified Glucksberg criteria were used (29). Chronic GvHD was assessed according to the 2014 NIH consensus statement (30).

### Statistical analysis

Categorical variables were expressed as the number and percentage of the group. Continuous variables were expressed as median with IQRs. Primary endpoints were OS and EFS since date of first HSCT. For OS, an event was defined as death of any cause. For EFS, an event was defined as graft failure, re-transplant, acute GVHD grade III or IV, moderate to severe chronic GVHD, or death of any cause, whichever occurred first. Patients without an event were censored at last follow-up. OS and EFS were determined using the Kaplan–Meier estimator, and subgroups were compared using the log-rank test. A multivariate Cox regression analysis was done to evaluate the association of pre-treatment JAK inhibitor use, alemtuzumab use, and HSCT before or after 2018 with survival. 2018 was the median year of transplant in our series. Statistical analyses were done using IBM SPSS Statistics version 29.0.0.0 and GraphPad Prism version 9.3.1.

### Online supplemental material

In Table S1, the follow-up information on the patients reported by Leiding et al. (18) is given. In Tables S2, S3, S4, and S5 and in Figs. S1 and S2, detailed information on the pretransplant features of the patients is given. Tables S6, S7, and S8; and Figs. S3, S4, and S5 have additional information on transplant procedures and outcome.

## Supplementary Material

Table S1 shows the follow-up data on patients previously reported by Leiding et al. (2018).

Table S2 shows the infections prior to first HSCT.

Table S3 shows the autoimmune disease prior to first HSCT.

Table S4 shows the antibody deficiencies prior to first HSCT.

Table S5 shows the performance status, HCT-CI, and number of hospitalizations prior to first HSCT.

Table S6 shows the GvHD prophylaxis or preventive treatment.

Table S7 shows the last evaluable chimerism result after first HSCT.

Table S8 shows the chimerism result per HSCT procedure.

## Data Availability

Data are available upon request due to legal/ethical reasons.
