# Supplementary material for: Improved outcome of HSCT in STAT1 gain-of-function disease following JAK inhibition bridging
Source: J Hum Immun. 2025 Jul 30;1(3):e20250027. doi: 10.70962/jhi.20250027 (PMC12551681; doi:10.70962/jhi.20250027)
Supplement: Table S1 — shows the follow-up data on patients previously reported by Leiding et al. (2018). [file jhi_20250027_tables1.docx]

**Supplemental Table 1. Follow-up data on patients previously reported by Leiding, et al. (2018)**

| **Patient number in previous report** | **Patient number in current report, follow-up data if available** |
| --- | --- |
| 1 | Alive, data not re-entered in current study because of HSCT date before 2010 |
| 2 | Alive, data re-entered in current study as #10 |
| 3 | Alive, data not re-entered in current study |
| 4 | Transplanted after 2010, data not re-entered in current study, but reported deceased since the initial report. |
| 5 | Died, data not re-entered in current study |
| 6 | Died, data re-entered in current study as #33 |
| 7 | Died, data re-entered in current study as #32 |
| 8 | Died, data not re-entered because of HSCT date before 2010 |
| 9 | Died, data not re-entered in current study |
| 10 | Alive, data not re-entered in current study |
| 11 | Alive, data re-entered in current study as #27 |
| 12 | Died, data re-entered in current study as #28 |
| 13 | Alive, data re-entered in current study as #7 |
| 14 | Died, data not re-entered because of HSCT date before 2010 |
| 15 | Died, data re-entered in current study as #30 |
