# Supplementary material for: Improved outcome of HSCT in STAT1 gain-of-function disease following JAK inhibition bridging
Source: J Hum Immun. 2025 Jul 30;1(3):e20250027. doi: 10.70962/jhi.20250027 (PMC12551681; doi:10.70962/jhi.20250027)
Supplement: Table S2 — shows the infections prior to first HSCT. [file jhi_20250027_tables2.docx]

**Supplemental Table 2. Infections prior to first HSCT**

|  | n | % |
| --- | --- | --- |
| **Infections** | 36 | 100% |
| Type of infection | | |
| Mucocutaneous fungal infections | 34 | 94% |
| Bacterial infections | 28 | 78% |
| Viral infection / reactivation | 21 | 58% |
| Invasive fungal infections | 9 | 25% |
| Mycobacterial infections | 4 | 11% |
| Parasitic infection (*G. lamblia*) | 1 | 3% |
| **Mucocutaneous fungal infections: pathogen** | | |
| *Candida* spp. | 35 | 97% |
| Dermatophytes | 4 | 11% |
| Dematophyte: site |  |  |
| Cutaneous mycosis | 3 | 8% |
| Onychomycosis | 2 | 6% |
| **Bacterial infections: pathogen** | | |
| *Staphylococcus aureus* | 11 | 31% |
| *Streptococcus* spp. | 9 | 25% |
| *Haemophilus influenzae* | 5 | 14% |
| *Pseudomonas aeruginosa* | 5 | 14% |
| *Clostridium difficile* | 2 | 6% |
| *Moraxella catarrhalis* | 1 | 3% |
| *Escherichia coli* | 1 | 3% |
| **Bacterial infections: site** | | |
| Lung | 16 | 44% |
| Skin | 16 | 44% |
| Other | 9 | 25% |
| Blood / disseminated | 3 | 8% |
| Adenitis | 1 | 3% |
| Brain / central nervous system | 1 | 3% |
| Unknown | 1 | 3% |
| **Viral infections** | | |
| CMV | 9 | 25% |
| Varicella-zoster | 6 | 17% |
| EBV | 4 | 11% |
| Molluscum contagiosum | 4 | 11% |
| Other | 4 | 11% |
| Warts | 3 | 8% |
| Herpes simplex | 2 | 6% |
| Influenza | 2 | 6% |
| Adenovirus | 2 | 6% |
| **Invasive fungal infections: pathogen** | | |
| *Aspergillus* spp. | 4 | 11% |
| *Candida* spp. | 3 | 8% |
| Mucormycosis | 1 | 3% |
| *Pneumocystis jirovecii* | 1 | 3% |
| Histoplasmosis | 1 | 3% |
| **Invasive fungal infections: site** | | |
| Lung | 6 | 17% |
| Blood / disseminated | 3 | 8% |
| ENT | 2 | 6% |
| **Mycobacterial infections: pathogen** | | |
| BCG strain | 1 | 3% |
| Unknown | 1 | 3% |
| *M. abscessus* | 1 | 3% |
| *M. avium* | 1 | 3% |
| *M. genavense* | 1 | 3% |
| **Mycobacterial infections: site** | | |
| Adenitis | 1 | 3% |
| Blood / disseminated | 1 | 3% |
| Lung | 1 | 3% |
