# Supplementary material for: Improved outcome of HSCT in STAT1 gain-of-function disease following JAK inhibition bridging
Source: J Hum Immun. 2025 Jul 30;1(3):e20250027. doi: 10.70962/jhi.20250027 (PMC12551681; doi:10.70962/jhi.20250027)
Supplement: Table S3 — shows the autoimmune disease prior to first HSCT. [file jhi_20250027_tables3.docx]

**Supplemental Table 3. Autoimmune disease prior to first HSCT**

|  | n | % |
| --- | --- | --- |
| Autoimmunity/inflammatory disease | 25 | 69% |
| Thyroid disease | 10 | 28% |
| Autoimmune cytopenia | 9 | 25% |
| Other | 9 | 25% |
| Colitis / enteropathy | 7 | 19% |
| Alopecia | 4 | 11% |
| Autoimmune hepatitis | 4 | 11% |
| Auto-antibodies without overt autoimmunity | 3 | 8% |
| Addison's disease | 2 | 6% |
| Celiac disease | 2 | 6% |
| Insulin Dependent Diabetes mellitus | 1 | 3% |
| Growth hormone deficiency | 1 | 3% |
| Systemic lupus erythematosus (SLE | 1 | 3% |
| Vitamin B12 deficiency anemia | 1 | 3% |
| Autoimmune cytopenia: type |  |  |
| Autoimmune anemia | 8 | 22% |
| Autoimmune neutropenia | 3 | 8% |
| Autoimmune thrombocytopenia | 2 | 6% |
