# Supplementary material for: Improved outcome of HSCT in STAT1 gain-of-function disease following JAK inhibition bridging
Source: J Hum Immun. 2025 Jul 30;1(3):e20250027. doi: 10.70962/jhi.20250027 (PMC12551681; doi:10.70962/jhi.20250027)
Supplement: Table S4 — shows the antibody deficiencies prior to first HSCT. [file jhi_20250027_tables4.docx]

**Supplemental Table 4. Antibody deficiencies prior to first HSCT**

|  | n | % |
| --- | --- | --- |
| Antibody deficiency | 23 | 64% |
| Low IgA | 11 | 31% |
| Low IgM | 10 | 28% |
| Low total IgG | 9 | 25% |
| Specific polysaccharide (SPAD) | 4 | 11% |
| Hypogammaglobulinemia or dysgammaglobulinemia, but specifics unknown | 4 | 11% |
| IgG2 subclass deficiency | 4 | 11% |
| IgG3 subclass deficiency | 1 | 3% |
| Other / Unknown | 2 | 6% |
