# Supplementary material for: Improved outcome of HSCT in STAT1 gain-of-function disease following JAK inhibition bridging
Source: J Hum Immun. 2025 Jul 30;1(3):e20250027. doi: 10.70962/jhi.20250027 (PMC12551681; doi:10.70962/jhi.20250027)
Supplement: Table S5 — shows the performance status, HCT-CI, and number of hospitalizations prior to first HSCT. [file jhi_20250027_tables5.docx]

**Supplemental Table 5. Performance status, HCT-CI and number of hospitalizations prior to first HSCT**

|  | **n** | **%** |
| --- | --- | --- |
| **Karnofsky (adults) or Lansky (children) performance status** |  |  |
| 100 | 8 | 22% |
| 90 | 10 | 28% |
| 80 | 5 | 14% |
| 70 | 6 | 17% |
| 60 | 3 | 8% |
| 50 | 2 | 6% |
| Unknown | 2 | 6% |
| **HCT-CI score** |  |  |
| 0 | 11 | 31% |
| 1 | 5 | 14% |
| 2 | 6 | 17% |
| 3 | 7 | 19% |
| 4 | 5 | 14% |
| 5 | 2 | 6% |
| **Number of hospitalizations** |  |  |
| More than 5 times | 20 | 56% |
| Between 2 - 5 times | 8 | 22% |
| Once | 4 | 11% |
| Yes, but frequency unknown | 1 | 3% |
| Unknown | 1 | 3% |
| None | 2 | 6% |
