# Supplementary material for: Improved outcome of HSCT in STAT1 gain-of-function disease following JAK inhibition bridging
Source: J Hum Immun. 2025 Jul 30;1(3):e20250027. doi: 10.70962/jhi.20250027 (PMC12551681; doi:10.70962/jhi.20250027)
Supplement: Table S6 — shows the GvHD prophylaxis or preventive treatment. [file jhi_20250027_tables6.docx]

**Supplemental Table 6. GvHD prophylaxis or preventive treatment**

|  | **n** | **%** |
| --- | --- | --- |
| **First HSCT** |  |  |
| none | 4 | 11% |
| Calcineurin inhibitor with methotrexate (with or without additional agents) | 9 | 25% |
| *Additional treatments: ATG (n=1), mycophenolate (MMF) (n=1), abatacept (n=1), steroids (n=2)* |  |  |
| Calcineurin inhibitor with mycophenolate (MMF) (with or without additional agents) | 13 | 36% |
| *Additional treatment: ATG (n=1)* |  |  |
| Calcineurin inhibitor with steroids | 5 | 14% |
| *Additional treatments: ruxolitinib (n=2, one started at day +39, one started after cell infusion), tocilizumab (n=1)* |  |  |
| Post-transplant cyclophosphamide | 3 | 8% |
| *Additional treatments: calcineurin inhibitor and mycophenolate (MMF) (n=2), calcineurin inhibitor only (n=1)* |  |  |
| Calcineurin inhibitor only | 1 | 3% |
| **Second HSCT** |  |  |
| Calcineurin inhibitor with methotrexate (with or without additional agents) | 3 | 75% |
| Additional treatment: ATG (n=1) |  |  |
| Post-transplant cyclophosphamide | 1 | 25% |
| *Additional treatment: calcineurin inhibitor, mycophenolate (MMF)* |  |  |
