# Supplementary material for: Improved outcome of HSCT in STAT1 gain-of-function disease following JAK inhibition bridging
Source: J Hum Immun. 2025 Jul 30;1(3):e20250027. doi: 10.70962/jhi.20250027 (PMC12551681; doi:10.70962/jhi.20250027)
Supplement: Table S7 — shows the last evaluable chimerism result after first HSCT. [file jhi_20250027_tables7.docx]

**Supplemental Table 7. Last evaluable chimerism result after first HSCT**

| Chimerism result | n | % |
| --- | --- | --- |
| *Full donor chimerism (>95% donor)* | *23* | *64%* |
| *Mixed chimerism* | *7* | *19%* |
| *Autologous reconstitution* | *4* | *11%* |
| *Not evaluable (death before engraftment or non-engraftment)* | *2* | *6%* |
