# Supplementary material for: Improved outcome of HSCT in STAT1 gain-of-function disease following JAK inhibition bridging
Source: J Hum Immun. 2025 Jul 30;1(3):e20250027. doi: 10.70962/jhi.20250027 (PMC12551681; doi:10.70962/jhi.20250027)
Supplement: Table S8 — shows the chimerism result per HSCT procedure. [file jhi_20250027_tables8.docx]

**Supplemental Table 8. Chimerism result per HSCT procedure**

| Chimerism result (per HSCT procedure, from first to last chimerism) | n | % |
| --- | --- | --- |
| *Full donor chimerism (>95% donor)* | *27* | *68%* |
| Persistent full donor chimerism (>95% donor) | 23 |  |
| Mixed chimerism to full donor chimerism (>95% donor) | 4 |  |
| *Mixed chimerism* | *8* | *20%* |
| Full donor chimerism (>95% donor) to mixed chimerism | 7 |  |
| Persistent mixed chimerism | 1 |  |
| *Autologous reconstitution or no engraftment* | *4* | *10%* |
| Complete autologous reconstitution (>95% recipient) | 2 |  |
| Mixed chimerism to complete autologous reconstitution (>95% recipient) | 2 |  |
| *Not evaluable (death before engraftment)* | *1* | *3%* |
